# Klippel – Feil Syndrome Associated with Congential Heart Disease Presentaion of Cases and a Review of the Curent Literature

**DOI:** 10.3889/oamjms.2015.022

**Published:** 2015-02-11

**Authors:** Ramush Bejiqi, Ragip Retkoceri, Hana Bejiqi, Naim Zeka

**Affiliations:** 1 *Division of Cardiology, Pediatric Clinic, University Clinical Center of Kosovo, Prishtina, Republic of Kosovo*; 2 *Main Center of Family Medicine, Prishtina, Republic of Kosovo*

**Keywords:** Klippel-Feil syndrome, short neck, low hairline, congenital heart disease

## Abstract

First time described in 1912, from Maurice Klippel and Andre Feil independently, Klippel-Feil syndrome (synonyms: cervical vertebra fusion syndrome, Klippel-Feil deformity, Klippel-Feil sequence disorder) is a bone disorder characterized by the abnormal joining (fusion) of two or more spinal bones in the neck (cervical vertebrae), which is present from birth. Three major features result from this abnormality: a short neck, a limited range of motion in the neck, and a low hairline at the back of the head. Most affected people have one or two of these characteristic features. Less than half of all individuals with Klippel-Feil syndrome have all three classic features of this condition.

Since first classification from Feil in three categories (I – III) other classification systems have been advocated to describe the anomalies, predict the potential problems, and guide treatment decisions. Patients with Klippel-Feil syndrome usually present with the disease during childhood, but may present later in life. The challenge to the clinician is to recognize the associated anomalies that can occur with Klippel-Feil syndrome and to perform the appropriate workup for diagnosis.

## Introduction

Klippel-Feil syndrome (KFS) is a congenital anomaly characterized by a defect in the formation or segmentation of the cervical vertebrae, resulting in a fused appearance. The clinical triad consists of short neck, low posterior hairline, and limited neck movement, although less than 50% of patients demonstrate all 3 clinical features [1]. However, despite the lack of accurate epidemiological data from Kosovo, KFS and variety are rare in children and adults on this region. KFS is usually non-malignant but causes significant morbidity, especially when is associated with anomalies in vital organs [2]. Discussion of unique aspects of etiology, diagnosis, and management in underserved regions of Kosovo may be important to raise awareness in health professionals, allows timely diagnosis, and improves management and prognosis. Here, we present four cases of patients with KFS and congenital heart disease, diagnosed at different age, surgical treatment and outcomes in Kosovo as a country with limited resources [3, 4].

Here, we report four cases in young Kosovars with KFS and heart abnormalities, clinical presentation, diagnosis, management, and outcomes of selected conditions in resources-limited settings.

## Case Reports

**Case 1**. A 2- days old male infant, weighing 3.5 kilograms, was noted to have bluish discoloration over the lips and periphery, with percutaneous oxygen saturation of 75%. The baby was born in Madrid from parents refugees from Kosovo during the war 1999. He required intubation following one apneic episode. Despite being ventilated on 100 % oxygen, pulse oximetry could not be raised above 80 %. Hence, a prostaglandin infusion was commenced. Physical examination showed a short neck, low hairline at the back of the head, and particularly restricted mobility of the upper spine. Cardiovascular and respiratory examination was normal.

**Figure 1:**
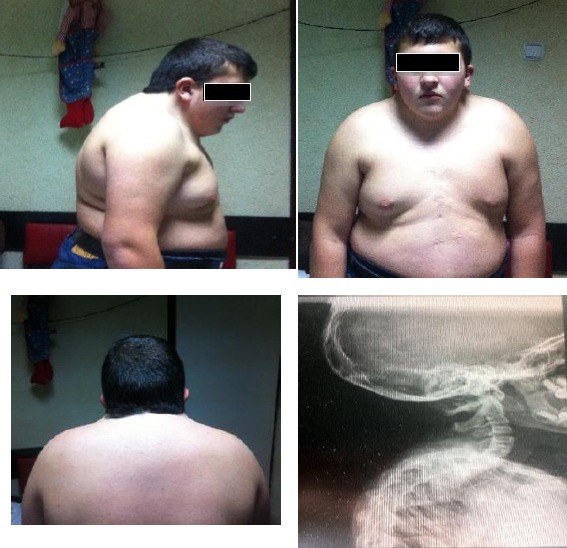
*Case 1. A 15-years-old boy diagnosed at delivery with Klippel – Feil syndrome. Note the short neck, low hairline and symmetric shoulders. (Scar on the thorax after surgical repair of total anomalous venous return)*.

An electrocardiogram showed tight atrial and ventricular hypertrophy. Chest X-ray showed cardiomegaly. Transthoracic echocardiography showed confluence of the all pulmonary veins above left atrium and draining together to the right atrium. The diagnosis of total anomalous venous return to the right atrium was confirmed. In the region of fosa ovale restrictive secundum atrial septal defect was presented with a predominantly right-to-left shunt.

Suspicion for KFS was indication for cervical radiography and multiple developmental anomalies of the upper cervical spine, consistent with Klippel-Feil syndrome. Antero posterior (AP) as well as lateral neutral, flexion, extension radiographs of the cervical spine demonstrate abnormal development of the upper cervical spine described as follows: There is at least a partial fusion of C2 and C3, the ossified portion of the dens is noted to be inferiorly displaced relative to the anterior arch of C1, the posterior elements of C1, C2 and likely C3 are hypoplastic/absent, and there is a defect in the lamina of the superior – most vertebral body which contains formed posterior elements. The fused vertebral bodies are small and also have an abnormal anterior convex configuration. On the flexion and extension radiographs and atlanto-axial, instability is identified. The skull projects over most of the cervical spine on the AP views so evaluation for vertebral bodies is limited. The posterior skull is noted to have a “beaten copper” appearance. A cervical rib is present on the right. The paravertebral soft tissues have normal appearance. The nasopharynx, oropharynx, and laryngopharynx are patent and clear.

Family history was normal for congenital heart disease, clefts, MR/DD, seizures, hearing loss, eye, Dx, diabetes, other birth defects, and pregnancy losses. Consanguinity is denied. Genetics examination confirmed new mutations in the GDF6

At the 7th day of birth, at the “Hospital Universitario La Paz” in Madrid surgical repair of the anomalous pulmonary venous return was done successfully and in the next day, in stable condition, was extubated. At the 15th day of life he was discharged on the only Captopril, 0.5mg/kg three times daily. Control echocardiography showed normal pulmonary venous return without obstruction and normal heart function.

**Case 2.** A 28-month-old girl, as the fourth child, from normal pregnancy and absolutely health parents, weighing 16.3 kg, during the routine pediatric examination systolic murmur was noted and for cardiological examination at tertiary level was referred. The child’s growth and development was completely normal. There was no sweating or fatigue during feeding or normal activities. Complete clinical and cardiological examination was obtained. Objective examination of other children is in normal limits.

**Figure 2:**
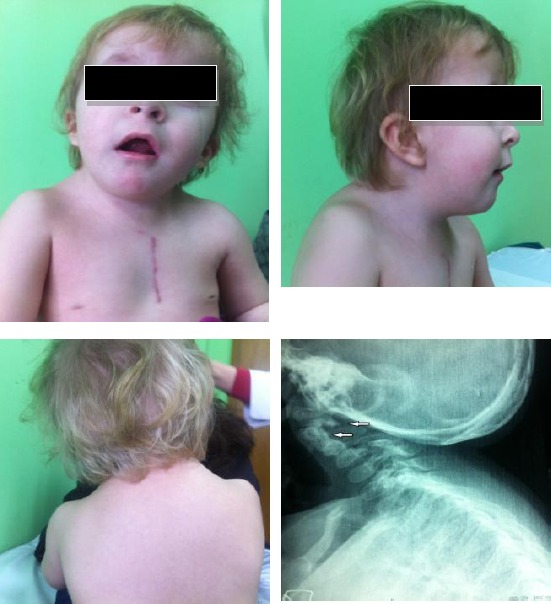
*Case 2. Patient with Klippel-Feil syndrome and anomaly of the occipito-cervical junction. The images show an elevated left shoulder due to a Sprengel anomaly, a short, webbed neck, and a low hairline. (Scar on the thorax after surgical repair of nonrestrictive atrial septal defect). X-ray shows occipito-cervical junction*.

During routine examination a short neck, low hairline at the back of the head and restricted mobility of the upper spine was noted. Anteroposterior (AP) and lateral in extension radiographs of the cervical spine demonstrate abnormal development of the upper cervical spine as a partial fusion of C2 and C3 and hypoplastic C1 spine. The fused vertebral bodies manifested an abnormal anterior convex configuration. Arterial blood gas was within normal limits. Clinical examination demonstrated: a quite precordium, normal first heart sound, short midsystolic murmur 2-3/6 degree on the apex and left sternal border, and single second heart sound. Electrocardiogram showed: normal sinus rhythm, left axis deviation, and incomplete bundle branch block. A chest radiogram revealed a normal cardiac silhouette.

The pulmonary vasculature was normal in appearance, and there were no infiltrates seen. Cross-sectional echocardiography demonstrated: normal systemic and pulmonary vein connection, big hole, diameter 12 mm, in the middle part of interatrial septum with nonrestrictive left to right flow. There was normal atrio-ventricular and ventriculo-arterial connection. By continuous Doppler waves and color Doppler trivial tricuspid regurgitation and hyperdynamic flow through the pulmonary, artery was noted. Family history for consanguinity and congenital heart disease is denied. At age of 18 month, in the absence of cardiac services in Kosovo, child was sent in the Santa Rosa Children’s Hospital (USA) and surgical correction of atrial septal defect has been successfully done. Also genetics examination confirmed new mutations in the GDF6 and GDF3 genes.

**Figure 3:**
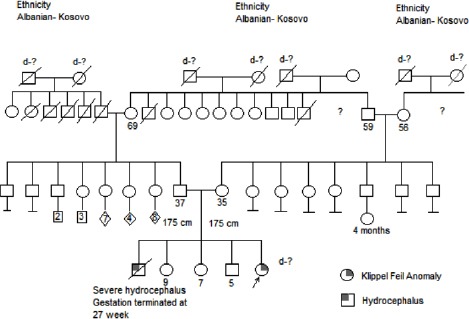
*Family tree of Case 2*.

**Case 3.** A 2-day-old male infant, weighing 2890 g., from the first and normal pregnancy, from both healthy parents, born in regional hospital, at the second day of life is presented with respiratory distress. Despite being ventilated on 100 % oxygen, pulse oximetry could not be raised above 85 %. Baby was transferred at tertiary level and a complete history and careful physical examination was obtained. Objectively a short, webbed neck, decreased range of motion in the cervical spine and a low hairline was noted and suspicion for KFS awakened. Anteroposterior (AP) and lateral radiographs of the cervical spine demonstrate abnormal development of vertebra C1, C2 and C3 with massive fusion of the cervical spine.

Echocardiography revealed situs solitus, levocardia, atrioventricular concordance and transposition of the great arteries with patent ductus arteriosus. At the third day of life balloon atrioseptostomy was performed and 7- 8 mm communication between atria was achieved. In the lack of cardio surgery services in Kosovo and financial impossibility of family to cover surgery abroad Kosovo, baby dies after 2 months as a consequence of congestive heart failure and respiratory infection.

**Figure 4:**
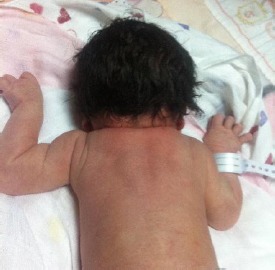
*A 2-days old neonate with Klippel-Feil syndrome. The image shows an elevated right shoulder due to a Sprengel anomaly, a short, webbed neck, and a low hairline*.

**Case 4.** An asymptomatic girl, aged 12 years, born to non-consanguineous parents and from normal pregnancy, so far with normal development, was referred for assessment of a recently discovered systolic cardiac murmur, grade at 2 from 6, and a best heard along the left parasternal border. During the clinical examination a very short neck, reduced bilateral neck movements, and a low hairline at the back of the head was noted. Radiographic examination showed fusion of C1 and C2 and C5-7 vertebrae. Transthoracic echocardiographic exami-nation, using Acuson Sequoia 256 machine, showed usual arrangement of the atrial appendages and thoraco-abdominal organs, with concordant atrio-ventricular and ventriculo-arterial connections. There was a slight enlargement of the right heart side. In the parasternal short-axis view thick pulmonary valve cusp with restricted systolic motion was noted. The main pulmonary artery was dilated. By Doppler and color Doppler turbulent flow through the pulmonary, artery was noted with max pressure of 68 mm of mercury.

## Incidence

The actual occurrence for the KFS syndrome is unknown; it is estimated to occur from 1 in 40,000 to 42,000 newborns worldwide. In addition, females seem to be affected slightly more often than males. Mutations in the GDF6 and GDF3 genes can cause Klippel-Feil syndrome. These genes provide instructions for making proteins that belong to the bone morphogenetic protein family, which is involved in regulating the growth and maturation (differentiation) of bone and cartilage.

**Figure 5:**
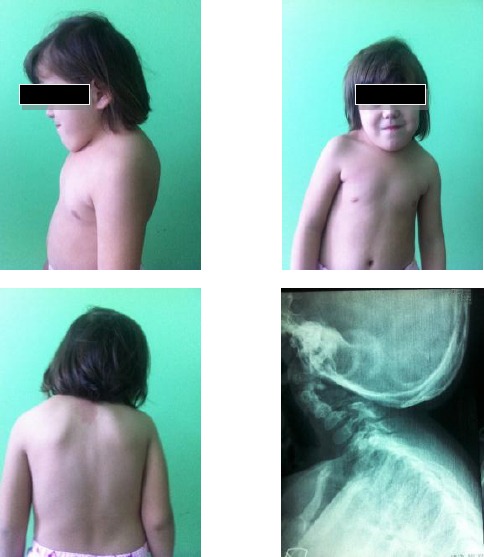
*A 12-years-old girl with Klippel-Feil syndrome and pulmonary stenosis. Note elevated left shuoldres and thoarx feformity*.

Additional forms of KFS include autosomal recessive KFS2 (214300), caused by mutation in the MEOX1 gene (600147) on chromosome 17q21, and autosomal dominant KFS3 (613702), caused by mutation in the GDF3 gene (606522) on chromosome 12p13. Sometimes this condition is inherited in an autosomal recessive pattern, which means both copies of a gene in each cell have mutations. However, in these cases, the gene involved is unknown. The parents of an individual with an autosomal recessive condition each carry one copy of the mutated gene, but they typically do not show signs and symptoms of the condition [5, 6].

## Etiology

The etiology of Klippel-Feil syndrome and its associated conditions is unknown. The syndrome can be presented with a variety of other clinical syndromes, including fetal alcohol syndrome, Goldenhar syndrome, anomalies of the extremities etc [5]. The occurrence of KFS in sibs and in consanguineous families suggests autosomal recessive inheritance of a form of the disorder. A close evaluation of the immediate family is indicated, because autosomal dominant inheritance with variable expression in affected individuals has been noted, although this is presumably rare. There is a strong association with congenital abnormalities of the genitourinary tract (30 – 40 %), including double collecting systems, renal aplasia and horseshoe kidney [6, 7].

## Discussion

These cases reported show several aspects of KFS in different ages of young Kosovars. They highlight common clinical presentation and etiology, as well as the challenges for the diagnosis and management of these clinical entities resources- poor settings.

KFS has diverse clinical presentation and etiology. The presenting symptoms and signs depend from the age at presentation (at birth – congenital type), on the primary disease (abnormality of the neck and restricted movement of the head and neck) as well as the associated anomalies, especially heart anomalies. Associated anomalies also may include abnormal curvature of the spine (scoliosis) and/or vertebral instability, spina bifida occulta, raised scapula (Sprengel’s deformity), absent rib(s) and other rib defects including cervical ribs, other skeletal abnormalities including skeletal malformations of the ear, nose, mouth and larynx including hearing impairment and cleft palate, malformations of the head and facial (craniofacial) area; anomalies of the urinary tract and/or kidney including absent or horse-shoe kidney; or structural abnormalities of the heart (congenital heart defects), mirror movements, webbing of the digits and digital hypoplasia. In addition, in some cases, neurological complications may result due to associated spinal cord injury. The challenge to the clinician is to recognize the associated anomalies that can occur with Klippel-Feil syndrome and to perform the appropriate workup for diagnosis [8-10].

Since the first classification from Feil in three categories (I – III) other classification systems have been advocated to describe the anomalies, predict the potential problems, and guide treatment decisions. Patients with Klippel-Feil syndrome usually are presented with the disease during childhood, but may present later in life [11, 12].

The disorder can be present at birth (congenital), but mild cases may go undiagnosed until later during life when symptoms worsen or first become apparent. For the first time, it has been described in 1912, independently from Maurice Klippel and Andre Feil. Three major features result from this abnormality: a short neck, a limited range of motion in the neck, and a low hairline at the back of the head [1, 13].

Clinical presentation is varied because of the different associated syndromes and anomalies that can occur in patients with Klippel-Feil syndrome. A complete history and careful physical examination may reveal some associated anomalies. Klippel-Feil syndrome involves the congenital fusion (failure of segmentation) of one or more cervical motion segments, and most patients have associated congenital anomalies of the cervical spine or other organs and systems. These anomalies may occur at the craniocervical junction (occipit-C2), the sub axial spine (below C2), or both [11]. Our cases have multiple developmental anomalies of the upper cervical spine consistent with Klippel-Feil syndrome. The posterior skull has a “beaten copper” appearance which may represent a normal variant or less likely reflective of gyral impression from increased intracranial pressure because it is only seen posteriorly [14-16].

Associated anomalies occur in the auditory system, neural axis, cardiovascular system, and the musculoskeletal system. Cardiovascular anomalies, mainly septal defects, were found in 7 patients in Hensinger’s series, with 4 of these individuals requiring corrective surgery [12, 17]. In our presentation all cases have a different CHD including those which from delivery threatening children’s life (first case with total anomalous pulmonary venous return).

People with Klippel-Feil syndrome may have other features in addition to their spine abnormalities. Some people with this condition have hearing difficulties, genitourinary abnormalities such as malformed kidneys, a type of birth defect that occurs during the development of the brain and spinal cord (neural tube defect), an opening in the roof of the mouth (cleft palate), or heart abnormalities [4, 6, 7]. Consultations from different specialties in Kosovo and in both 1^st^ and 2^nd^ case during the surgical intervention abroad, other anomalies have been eliminated. Careful examinations of specialist exclude anomalies in other organs and systems. Radiographs and MRI of the thoracic and lumbosacral spine are obtained and other anomalies have been excluded.

**Table 1:** Review of literature.

Lead author	Year	No. of patients	Siblings
Lubs [18]	1963	2 from11	parents consanguineous
Juberg and Gershanik [19]	1976	1	parents consanguineous
Chemke [20]	1980	1	parents no consanguineous
Da-Silva [21]	1982	4 sibs (7f/5m)	parents consanguineous
Fragoso [22]	1982	1	parents no consanguineous
Clarke [23]	1998	Family	autosomal recessive
Erol [24]	2004	1	parents consanguineous
Ohashi [25]	1992	1	de novo balanced translocation
Ramush [26]	2013	1	parents no consanguineous

Affected individuals may have underde-veloped shoulder blades that sit abnormally high on the back, a condition called Sprengel deformity [13]. In three of our cases we have found underdeveloped shoulder where two of them have an elevated left shoulder and the other one has the right.

Lateral flexion-extension radiographs of the cervical spine should be performed on all patients to determine the motion of each open interspace. Clinically, flexion-extension is often maintained if a single functioning open interspace is maintained. Those with hyper mobility of the upper cervical segment are at risk of developing neurologic impairment. Affected individuals with hyper mobility of the lower cervical segment are at increased risk for degenerative disk diseases and should be treated symptomatically [12, 16].
